# Future Temperature‐Related Deaths in the U.S.: The Impact of Climate Change, Demographics, and Adaptation

**DOI:** 10.1029/2023GH000799

**Published:** 2023-08-15

**Authors:** Jangho Lee, Andrew E. Dessler

**Affiliations:** ^1^ Department of Atmospheric Sciences Texas A&M University College Station TX USA

## Abstract

Mortality due to extreme temperatures is one of the most worrying impacts of climate change. In this analysis, we use historic mortality and temperature data from 106 cities in the United States to develop a model that predicts deaths attributable to temperature. With this model and projections of future temperature from climate models, we estimate temperature‐related deaths in the United States due to climate change, changing demographics, and adaptation. We find that temperature‐related deaths increase rapidly as the climate warms, but this is mainly due to an expanding and aging population. For global average warming below 3°C above pre‐industrial levels, we find that climate change slightly reduces temperature‐related mortality in the U.S. because the reduction of cold‐related mortality exceeds the increase in heat‐related deaths. Above 3°C warming, whether the increase in heat‐related deaths exceeds the decrease in cold‐related deaths depends on the level of adaptation. Southern U.S. cities are already well adapted to hot temperatures and the reduction of cold‐related mortality drives overall lower mortality. Cities in the Northern U.S. are not well adapted to high temperatures, so the increase in heat‐related mortality exceeds the reduction in cold‐related mortality. Thus, while the total number of climate‐related mortality may not change much, climate change will shift mortality in the U.S. to higher latitudes.

## Key Points

- Temperature‐related deaths in the U.S. will increase by a factor of 5 with 3°C of warming, mostly due to aging and increasing population
- Impact of climate change is not significant until 3°C of global warming, and it depends on the level of adaptation beyond that point
- Temperature‐related deaths will shift Northward, due to increasing heat‐related deaths in poorly heat‐adapted Northern cities

## 1 Introduction

The relationship between temperature and human mortality has been the subject of many previous studies (Berko, 2014; Bobb et al., 2014; Demoury et al., 2022; Dimitrova et al., 2021; Gasparrini & Armstrong, 2011; Gasparrini et al., 2015b; Guo et al., 2011; Kalkstein & Greene, 1997; Ma et al., 2015; Yi & Chan, 2015; Zhang et al., 2016). Previous studies have projected future temperature‐related mortality covering different regions, such as global major cities in the U.S. (Anderson et al., 2018; Barreca, 2012; Jackson et al., 2010; Knowlton et al., 2007; Lo et al., 2019; Petkova et al., 2017; Wang et al., 2016; Weinberger et al., 2017), Europe (Hajat et al., 2014; Martínez‐Solanas et al., 2021; Muthers et al., 2010), or Asia (Lee & Kim, 2016; Yang et al., 2021). Using historical data sets, previous studies have found that temperature and mortality show a V‐shaped curve, where mortality increases as temperatures become very hot or very cold (Berko, 2014; de Schrijver et al., 2022; Dimitrova et al., 2021; Gosling et al., 2009; Vardoulakis et al., 2014).

Prior research has found that climate change will significantly impact future temperature‐related mortality; however, these impacts vary depending on location and the implementation of adaptation and mitigation strategies (Gasparrini et al., 2015b, 2017; Lee and Kim, 2016; Martínez‐Solanas et al., 2021). For example, Gasparrini et al. (2017) discovered that regions experiencing less intense warming will witness a decline in temperature‐related deaths, primarily due to a substantial reduction in cold‐related deaths. Conversely, areas subjected to intense warming will see a rise in temperature‐related deaths, driven by a considerable increase in heat‐related deaths.

A particular issue we explore in this paper is the impact of demographics. Older populations are known to be more vulnerable to temperatures extremes (Anderson et al., 2018; Åström et al., 2013; Barnett, 2007; Bobb et al., 2014; de Schrijver et al., 2022; Hintz et al., 2018; Lee & Kim, 2016; Lin et al., 2011; Yi & Chan, 2015; Zhang et al., 2016), and since population is projected to both age and grow globally, the compound effect of demographic and population changes will increase temperature‐related mortality (Li et al., 2016; Marsha et al., 2018). Previous studies included demographic and population change in their projection (Deschênes & Greenstone, 2011; Deschenes & Moretti, 2009; Hajat et al., 2014; Jenkins et al., 2014; Lee & Kim, 2016; Li et al., 2016; Petkova et al., 2017; Vardoulakis et al., 2014), mostly using population projections from shared socioeconomic pathways (SSPs) (Hauer, 2019). Utilizing SSPs, prior research has found that the combined effects of a growing and aging population will exacerbate the increase in temperature‐related mortality (Anderson et al., 2018).

It is also clear that people will take actions to head off the impacts of extreme temperatures (Barreca et al., 2016; Carson et al., 2006; Davis et al., 2003; Folkerts et al., 2020; Fouillet et al., 2008; Gasparrini et al., 2015a; Kyselý & Plavcová, 2012). However, such adaptation takes resources, which many people do not have. Consequently, how well this can be done is an uncertainty that any analysis of future temperature‐related mortality must address.

There are few ways to incorporate adaptation into estimate future temperature related deaths. Previous studies simply shifted the temperature‐mortality relationship to warmer temperatures (Folkerts et al., 2020; Gosling et al., 2009; Jenkins et al., 2014), extrapolated the historical trends of temperature‐mortality relationship (Muthers et al., 2010; Petkova et al., 2017), or adjusted the slope of temperature‐mortality relationship (Jenkins et al., 2014). In this study, we adopt the “analogue city” approach (Heutel et al., 2021; Kalkstein & Greene, 1997; Knowlton et al., 2007), wherein the temperature‐mortality model of a city currently experiencing a warmer climate is utilized to establish a mortality model for a presently cooler city under future warming conditions. For example, Knowlton et al. (2007), posited that New York in the 2050s would exhibit a temperature‐mortality relationship akin to that of Washington and Atlanta in 1973–1994, as the projected temperatures for New York in the 2050s resemble those of Washington and Atlanta during 1973–1994 period. We have developed a new way of incorporating analog cities into our analysis and we use that to estimate the impact of adaptation.

In this paper, we consider all three of the factors that will impact future temperature‐related mortality: climate change, population and demographics change, and adaptation to determine how important each factor is for mortality in the U.S. urban regions.

## 2 Temperature‐Mortality Relationship

**Figure 1:**
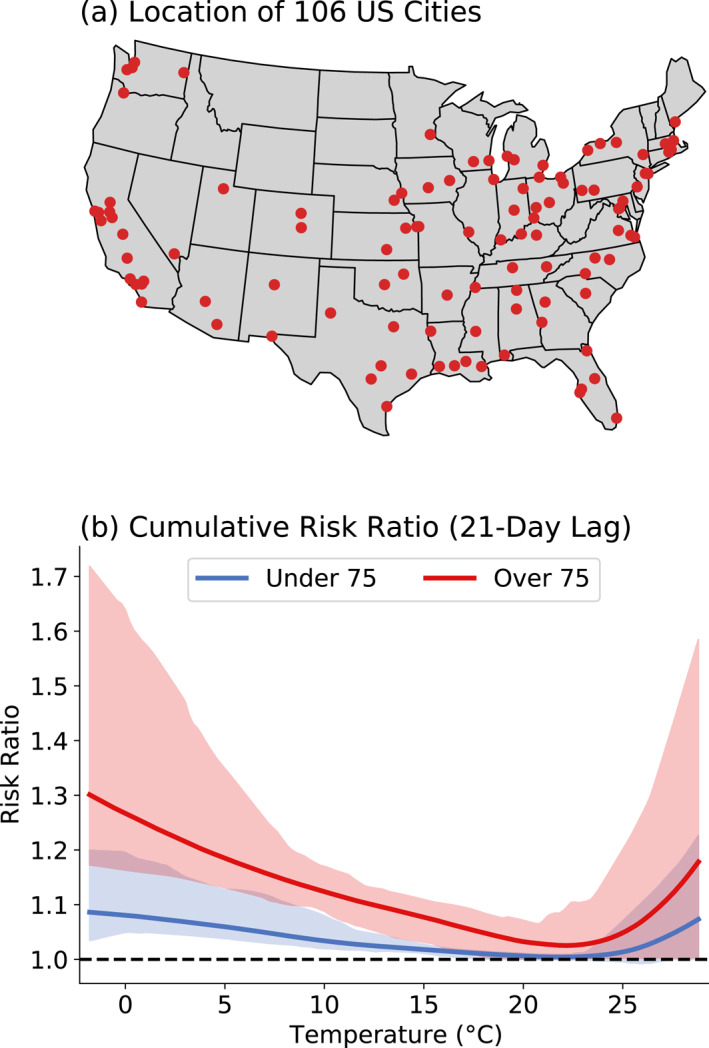
(a) Location of 106 cities used in this study. (b) Risk ratio (RR) of under/over 75 age groups, averaged for all cities in this study. Shaded regions show the 5th percentile to 95th percentile range of RR curve for all cities. RR is the number of deaths at each temperature divided by the number of deaths at the curve's minimum (the minimum mortality temperature), around 22°C.

Mortality data from the National Morbidity Mortality Air Pollution Study (NMMAPS) (Samet et al., 2000) contain the number of daily non‐accidental deaths, stratified by age group (<65, 65–75, and >75). We combine the two younger age groups into a single category for ages <75. By this, we have a similar number of deaths in the under 75 and over 75 age groups (49% and 51%, respectively). Data are collected from 106 large U.S. cities (Figure 1a), which contains 65% of the population in the U.S., and cover the period from 1987 to 2000. Population data are also included in NMMAPS, which come from the National Center for Health Statistics. More recent data exhibit limitations, such as being available only on a monthly basis or aggregated at a broader regional level (e.g., state level), which makes them unsuitable for the purpose of this analysis.

Historical hourly 2‐m air temperatures from ERA‐5 Land reanalysis (Muñoz‐Sabater et al., 2021) are averaged to obtain daily average temperatures. The data have a horizontal resolution of 0.1° × 0.1° and the average of the nine grid points nearest to the center of each city are used to represent the daily average temperature of the city. Nine grid points encompass an area of approximately 20 × 20 km in the city center, providing a reasonable representation of the city's temperature.

Following the framework of Gasparrini et al. (2015a, 2015b) we use a Distributed Lag Non‐Linear Model (DLNM) to describe the association between temperature and mortality. We model the daily number of deaths as a function of daily average temperature separately for each city and age group (under 75 and over 75). An important advantage of the DLNM is that it captures the lagged effect of temperature, where consecutive extreme days results in higher mortality than a single‐day event (Gasparrini & Armstrong, 2011; Wang et al., 2016).

Previous studies reported that the impact of a hot day can extend for up to 3 days, while the impact of a cold day could extend 21 days (Demoury et al., 2022; Dimitrova et al., 2021). Therefore, we include lags of up to 21 days in the DLNM model. We also include the day of week to account for the weekly cycle, day of year for the annual cycle, and year for the long‐term trend. A detailed explanation of the DLNM model used in this study can be found in Section S1 in Supporting Information S1. Additionally, the sensitivity of including ozone as another confounder in this analysis (Porter and Heald, 2019; Ren et al., 2008) is also discussed in Section S1 in Supporting Information S1.

## 3 Historical Temperature‐Related Mortality

Figure 1b summarizes the mortality risk as a function of temperature, averaged over all cities (curves for 25 most populated cities can be found in Figure S1 in Supporting Information S1). The quantity plotted here, the cumulative risk ratio (RR), is the number of deaths at each temperature divided by the number of deaths at the minimum mortality temperature (MMT), after summing the RR at each lag, up to 21 days. Our curve is similar to those found in previous work (Gasparrini et al., 2015a, 2015b, 2017; Guo et al., 2011; Lin et al., 2011; Ma et al., 2015; Yi & Chan, 2015; Zhang et al., 2016).

With the regression models for each city and age group, we calculate the number of temperature‐related excess deaths in the NMMAPS data in two steps. First, we define baseline deaths, which is the number of deaths at the MMT, calculated by averaging the number of deaths at temperatures around MMT (±0.5°C). With this baseline death value, we then calculate the number of deaths in each city using observed temperatures and the mortality‐temperature curves.

**Figure 2:**
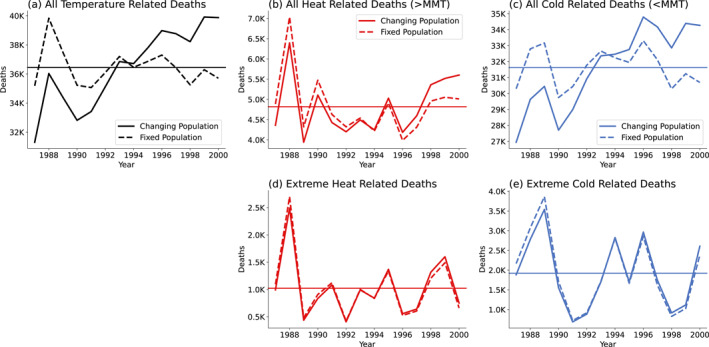
Time series of temperature‐related deaths across all 106 cities. (a) The solid line represents total temperature‐related deaths, while the dashed line indicates temperature‐related deaths with a fixed population (average population from the 1987–2000 period). (b) Similar to (a), but for heat‐related deaths. (c) Similar to (a), but for cold‐related deaths. (d) Time series of deaths occurring in temperatures above the 97.5th percentile. (e) Similar to (d), but for temperatures below the 2.5th percentile.

There are an average of 36,444 temperature‐related deaths per year between 1987 and 2000 (solid line in Figure 2a). There is a clear trend over this period, and we can remove the effect of population changes by dividing the number of excess deaths by the population in each year, and then multiplying by the average population over the period. Doing this removes most of the trend (dashed line in Figure 2a).

We will separate temperature‐related deaths occurring above and below MMT, which by convention we refer to as heat‐ and cold‐related deaths. We estimate there are an average of 4,819 heat‐related deaths per year and 31,625 cold‐related deaths. This is consistent with previous work that also found that most of the deaths were due to cold, rather than heat (Vardoulakis et al., 2014). Upon examining the age distribution of temperature‐related deaths in each city, we found that, on average, 75.3% of deaths are attributed to the older age group (over 75), with an inter‐city standard deviation equal to 6.2%. The older age group accounts for 75.6% (1*σ* = 4.6%) of heat‐related mortality and 75.1% (1*σ* = 6.9%) of cold‐related mortality, despite comprising only 5.1% (1*σ* = 1.3%) of the population. This fact that temperature‐related mortality is mainly in the older age groups will be important later in the paper.

Although 86% of temperature‐related deaths are due to cold‐related mortality (4,819 heat‐related deaths, 31,625 cold‐related deaths), most of these “cold‐related” deaths occur at temperatures only slightly below the MMT, which is typically around 22°C. For example, 26% of cold‐related mortality is occurring within 5°C of the MMT, with an inter‐city standard deviation of 11%. While the risk of temperature‐related death at these pleasant temperatures is low, their frequent occurrence still contributes to a significant number of deaths, a point also highlighted by Gasparrini et al. (2015a, 2015b).

This led us to investigate mortality resulting from extreme heat and cold. For each city, we selected days with temperatures exceeding the 97.5th percentile (calculated from the 1987–2000 temperatures) and falling below the 2.5th percentile (Gasparrini et al., 2015b), which we refer to as extreme heat‐ and cold‐related deaths (Figures 2d and 2e). When considering all cities, there are, on average, 1,022 deaths per year attributable to extreme heat and 1,922 to extreme cold, representing 21% and 6% of total heat and cold‐related deaths, respectively. This indicates that heat‐related deaths tend to be more concentrated around extreme heat, while cold‐related deaths exhibit a less pronounced skew toward extreme cold.

## 4 Adaptation

We know that people will adapt as much as possible to extreme heat. However, adaptation takes resources, and it is unclear the extent to which successful adaptation is achievable. Therefore, we analyze two limiting adaptation scenarios. In the first scenario, we assume no further adaptation, with the relative risk (RR) curve for each city remaining constant based on the 1987–2000 mortality data as the climate warms (Figure S1 in Supporting Information S1). This is the no‐adaptation scenario.

**Figure 3:**
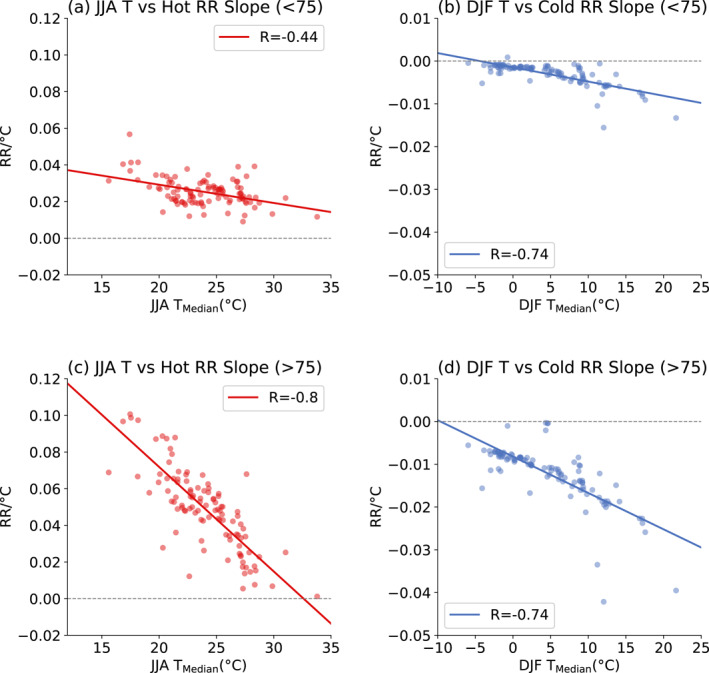
Relationship between the slope of each city's RR curve and that city's climate. (a) Relationship between slope of RR curve above minimum mortality temperature (MMT) (hot RR slope) for under 75 age groups and the JJA median daily temperature. The points represent individual cities, and the line is a linear regression fit. (b) Same as (a), but for slope of RR curve below MMT (cold RR slope) and the median DJF temperature. (c, d) Same as (a, b), but for over 75 age groups.

Our second scenario represents a strong adaptation case, in which cities adapt effectively to the warmer temperatures. We begin this calculation by quantifying how well adapted each city is in the 1987–2000 climate by calculating the linear slope of each city's cumulative RR versus temperature for temperatures above the MMT (hot RR slope) and slope of RR below the MMT (cold RR slope). While the RR curves are not linear, the linear fit is a metric for how steeply the curve rises. We do this fit separately for each age group. Figures 3a and 3c show the hot RR slope of all of the cities regressed against median of daily average temperatures of that city's hot season (June, July, and August, JJA) over the 1987–2000 period. Figures 3b and 3d show the cold RR slope regressed against the median daily average temperature of the cold season (December, January, and February, DJF).

There is a clear anti‐correlation between the RR slopes and seasonal temperatures. Cities with warmer summers are less vulnerable to heat‐related mortality (hot RR slopes are lower, Figures 3a and 3c), while cities with colder winters are less vulnerable to cold‐related mortality (cold RR slopes are lower, Figures 3b and 3d). One can also think of these fit lines in Figure 3 as measures of how humans adapt to changes in climate (Gasparrini et al., 2015b; Heutel et al., 2021; Kalkstein & Greene, 1997; Knowlton et al., 2007).

The regression lines in Figure 3 between temperature and slope of cities' RR curves allows us to parameterize how the RR curves change as temperatures warm. In our strong adaptation assumption, we assume that each cities' RR slope evolves following the regression lines in Figure 3, using each city's temperature over the previous 10 years to estimate that year's RR slope. Hot cities such as Miami, Houston, and Phoenix will warm past the data points in Figure 3, that is, they lack suitable analogs in a warmer climate. For those cities, we simply extrapolate the regression lines into the warmer climates, although we do not allow the slope to decline below zero. Cities that are already hot and well‐adapted to high temperatures are affected by this constraint. At 3°C global warming, the hot side RR slope (Figures 3a and 3c) falls below zero in Austin (TX), El Paso (TX), Houston (TX), Los Angeles (CA), Miami (FL), Phoenix (AZ), San Antonio (TX), and Tucson (AZ) (eight cities). The impact of constraining hot side RR slope not to fall below zero will be discussed later in the paper. We account for adaptation on the cold side by scaling the cold RR slope by 18.5% of the ratio used to scale the hot RR slope (18.5% represents the ratio of the hot‐side to cold‐side fits shown in Figure 3). Having established the RR slopes for both no adaptation (points in Figure 3) and strong adaptation (modified RR slope due to temperature metric change), we can estimate the impact of adaptation on temperature‐related mortality. A detailed example of incorporating adaptation is provided in Section S3 in Supporting Information S1.

Another way we could have included adaptation is to shift the MMT toward warmer temperatures as the climate warms. This will reduce deaths due to warm temperatures, but how it affects cold mortality depends on what assumption is made for how the cold side of the RR curve evolves as the MMT shifts. If one just translates the RR curve as the MMT shifts, then cold mortality increases, largely offsetting the benefits of reduced warm mortality. Other assumptions are of course possible. Since we don't know what the best assumption is, we decided to not adjust the MMT as the climate warms. In the end, our preliminary calculations suggest that this decision does not impact the conclusions of the paper.

## 5 Future Temperature‐Related Deaths

For our projection of future temperature‐related mortality, we utilize historical and RCP 8.5 scenario runs from NA‐CORDEX (Mearns et al., 2017), which contain bias‐corrected outputs of regional climate model runs over North America, using boundary conditions from global climate models (GCM). Twelve combinations of GCMs and RCMs are used in this study, and these are summarized in Table 1. Historical simulations cover the period from 1950 to 2005 and RCP 8.5 simulations cover 2006 to 2099. The NA‐CORDEX data have a 0.22° × 0.22° horizontal resolution, so we use the four grid points nearest to each city to represent the city's temperature. This covers approximately a 22 × 22 km area in the city center, which is consistent with the ERA‐5 temperatures used to develop the temperature‐mortality model.

**Table 1:** Description of NA‐CORDEX Members Used in This Study

Scenario	Global climate model	Regional climate model	Bias‐correction
Historical + RCP 8.5	CanESM2	CanRCM4	MBCn using Daymet
		CRCM5‐UQAM	
	GEMatm‐Can	CRCM5‐UQAM	
	GEMatm‐MPI	CRCM5‐UQAM	
	GFDL‐ESM2M	RegCM4	
		WRF	
	HadGEM2‐ES	RegCM4	
		WRF	
	MPI‐ESM‐LR	CRCM5‐UQAM	
		RegCM4	
		WRF	
	MPI‐ESM‐MR	CRCM5‐UQAM	

We validate the NA‐CORDEX ensemble by predicting temperature‐related deaths in 1987–2000 period. We do this by plugging temperatures from the NA‐CORDEX ensemble for each city over that period into that city's regression model. The average number of temperature‐related deaths estimated using NA‐CORDEX temperatures is 36,675 (inter‐model 95% CI = 36,189–37,231), in which 5,067 (95% CI = 4,666–5,332) deaths are heat‐related, and 31,608 (95% CI = 31,111–32,331) are cold‐related. Using ERA‐5 temperatures, we estimated 36,444, 4,819, and 31,625 deaths, respectively. This provides some confidence in the NA‐CORDEX temperature fields.

For future temperature‐related mortality predictions, we also need predictions of population and demographics. For this, we use the SSP5 scenario, a fossil‐fueled development scenario, which corresponds with the RCP8.5 emissions scenario (Juzbašić et al., 2022; Rohat et al., 2021; Roy et al., 2021), which is driving the NA‐CORDEX models. We use data from Hauer (2019), which contains county‐level estimates of population and demographics at 5‐year intervals from 2020 to 2100. To convert the county‐level estimate to the city level, we extract counties containing each city in our analysis. 74% of the cities are within one county, and for these we assume that the city's population remains a constant fraction of the county's population. For cities that are in multiple counties, we sum the population of all counties that include the cities. Because the counties and city do not perfectly overlap, we take historical demographic data from 2020 and SSP5 data from 2020 and estimate the fraction of the total counties' population living in the city, and assume that fraction is constant over the century. From this, we come up with time series of population estimates in two age groups for each city in the coming century. A summary of population and demographic projections, as well as sensitivity due to choice of socioeconomic pathways can be found on the Section S4 in Supporting Information S1.

To estimate future deaths, we plug NA‐CORDEX temperatures for the 21st century for each city into that city's regression model (Figure S1 in Supporting Information S1) and then use population and demographic information to convert the RR values to temperature‐related mortality numbers.

RCP 8.5 emissions will likely exceed actual emissions, so we plot estimated mortality as a function of global average surface temperature (relative to the 1850–1859 period, before significant human‐induced warming occurred). We estimate global average warming in each year of the NA‐CORDEX from averages of the four GCM included in NA‐CORDEX: CanESM2 (5 ensemble members) (Chylek et al., 2011), GFDL‐ESM2M (1 run) (Dunne et al., 2020), HadGEM2‐ES (3 ensemble members) (Collins et al., 2011), and MPI‐ESM (100 ensemble members) (Maher et al., 2019) with historical and RCP 8.5 forcing. To do this, we first average the ensemble members of each climate model and then average those to come up with a single global average temperature time series. This gives us the global average warming of 0.83 in 2000 and 1.37°C in year 2022, close to observed values in ERA‐5. We did not incorporate other emission scenarios (e.g., RCP 4.5 and RCP 2.6) in this study because the NA‐CORDEX database lacks sufficient model runs for those scenarios.

Most cities will warm more than the global average. A global temperature increase of 3°C relative to pre‐industrial times corresponds to 2°C warming above the 2012–2022 period. The 106 major cities analyzed here are expected to warm by 2.41°C relative to 2012–2022, 20% more than the global average. Cities further from the coast and at higher latitude are expected to warm up more. The top three cities with the highest projected temperature increases are Minneapolis, MN (0.96°C larger than the global average), Milwaukee, WI (0.88°C), and Muskegon, MI (0.86°C). Detailed city‐by‐city data are available in Table S2 in Supporting Information S1.

**Figure 4:**
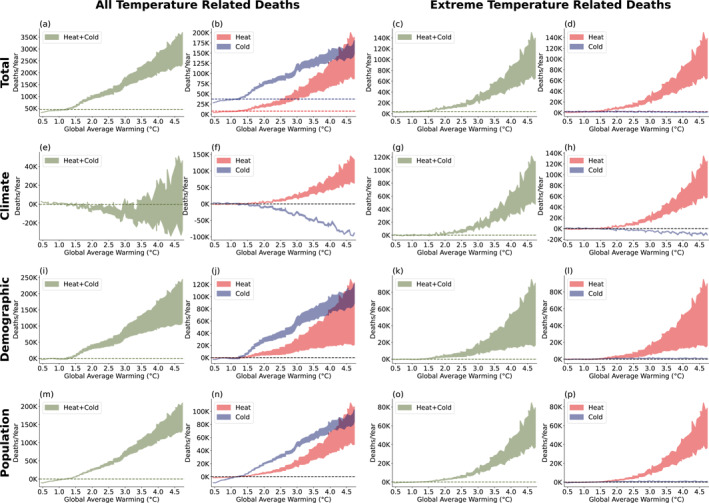
Estimates of future temperature‐related deaths as a function of global average warming. (a–d) Future temperature‐related deaths incorporating all factors: climate, demographics, and population. Upper limit of shaded region represents no‐adaptation scenario, while the lower limit represents the adaptation scenario. (a) All temperature related mortality, (b) heat‐ and cold‐related deaths, (c) mortality due to extreme temperatures, (d) mortality due to extreme heat and cold. Lower rows follow the same pattern as (a–d), but considering only climate change (e–h), demographics change (i–l), and population change (m–p). In all panels, dashed lines represent the average of the current value (2011–2020).

With future climate projections from NA‐CORDEX, future population and demographics projection from SSP5, and our two adaptation scenarios, we calculate future temperature‐related mortality (Figures 4a–4d). Looking at total temperature‐related deaths with no adaptation, we find that there are 45,800 deaths annually between 2011 and 2020 (1.16°C warming) and that is projected to grow to 200,000 annually with 3°C of global average warming, with both heat‐ and cold‐related deaths increasing (Figures 4a and 4b). Adaptation will decrease this number, reducing the increase of temperature‐related mortality at 3°C by about 28%. There are 12,500 deaths annually between 2011 and 2020 due to extreme temperatures (Figures 4c and 4d), which is projected to increase to 63,000 annually at 3°C, a proportionally larger increase than all‐temperature deaths.

We now decompose the increase in temperature‐related mortality into contributions from climate change, demographics change, and population change. To estimate the impact of each of these terms, we repeat the mortality calculation with that term fixed and then subtract the values obtained from the calculation with all terms varying.

To estimate the impact of climate change, we fix climate by repeating the daily temperature of recent years (2011–2020) for the entire period (1987–2100) and then subtracting the resulting temperature‐related mortality from the all‐factor calculation. For the no‐adaptation case, lives saved by less cold balances the lives lost due to more hot temperatures until about 3°C. Above that, the increase in heat‐related mortality overwhelms and total mortality rises rapidly. For the strong adaptation scenario, temperature‐related mortality decreases at all temperatures. Allowing the RR slope to decrease below zero results in a 50% reduction in the number of temperature‐related deaths in eight cities that were restricted by the constraint, corresponding to an annual decrease of 2,192 deaths at a 3°C warming level. This represents a 1.1% decrease in total temperature‐related deaths nationally, indicating that it has a minimal effect on our overall findings.

We also find that temperature‐related mortality in response to extreme temperatures will increase at all levels of warming (Figures 4g and 4h). This tells us that most of the lives saved in a warming world are due to a reduction in moderate cold temperatures.

Next, we look at impact of demographics (Figures 4i–4l) by performing a fixed‐demographics calculation that fixes the ratio of under/over 75 population to the 2011–2020 average and then subtracting this from the all‐factor calculation. We find that the aging of our population drives an enormous increase in deaths (Figure 4i) due to the older age group being more vulnerable to temperature‐related mortality (Figure 4j).

Finally, we calculate the impact of population (Figures 4m–4p) by fixing population at the 2011–2020 average value and subtracting the results from the all‐factor calculation. As the population increases, the total number of deaths also increases.

Comparing the three contributing terms, we find that changes in demographics and population are the most important driver of future mortality, and then climate change. This likely reflects the enormous investments in adaptation that have already been made (e.g., nearly 100% air conditioner penetration in cities like Phoenix and Houston). These results stand in contrast to analyses of poorer countries, which show large increases in temperature‐related mortality as the climate warms in the future (Carleton et al., 2022).

## 6 The Spatial Pattern of Temperature‐Related Deaths

**Figure 5:**
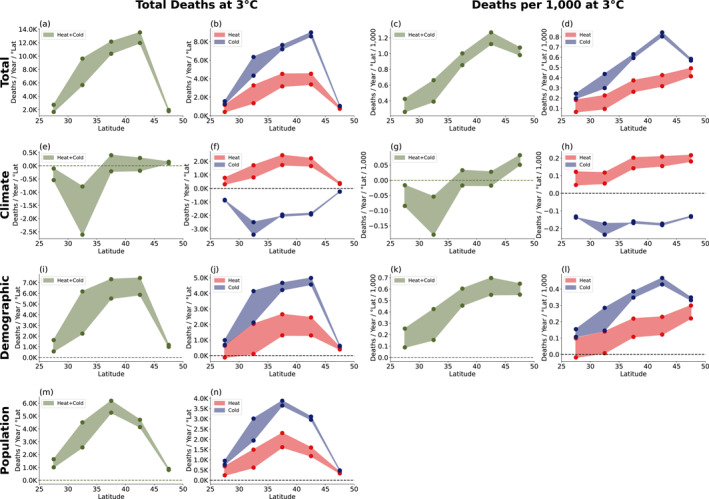
Meridional distribution of temperature‐related deaths in 3°C world. (a) Number of temperature related deaths in 3°C world. The upper limit of the shaded region represents no‐adaptation scenario, while the lower limit represents the adaptation scenario. (b) Same as (a), but for heat‐ and cold‐related deaths. (c, d) Same as (a, b), but per capita (each bin has been divided by population in that bin). (e–h) Contribution of climate change to mortality, (i–l) contribution of demographic changes to mortality, (m–n) contribution of changes in population.

We now analyze the spatial distribution of heat‐related mortality. We focus on the meridional variations in number of deaths at 3°C global average warming, approximately business‐as‐usual warming for 2100. We find that most of the temperature‐related deaths occur between 40° and 45°N (Figure 5a). Analyzing per capita deaths, we find they are also weighted toward higher latitudes (Figure 5c).

Looking at the climate contribution (Figures 5e and 5g), we see that climate change shifts mortality poleward: decreasing deaths at lower latitudes and potentially increasing them (depending on adaptation) at higher latitudes. This occurs because Southern cities in the U.S. are already well adapted to heat (Figure 3), so further warming does not add significantly to heat‐related deaths. However, these Southern cities do experience a decline of cold‐related deaths, leading to a net reduction in temperature‐related mortality. Northern cities, on the other hand, are less adapted to heat, so they experience large increases in heat‐related mortality, which exceeds the decline in cold‐related mortality (Carleton et al., 2022; Knowlton et al., 2007).

The impact of adaptation is particularly pronounced between 30° and 35°N (Figure 5e) due to demographics. Currently, the 30°–35°N region is the second youngest region (percentage of over 75 age group = 5.29%); when the Earth reaches 3°C of global average warming, it will be the oldest region (17.32%). Since the older age group is both more vulnerable to high temperatures and more sensitive to adaptation (Figure 3), adaptation will have a large impact on mortality over this latitude range.

We have made similar plots for extreme heat‐ and cold‐related deaths (mortality in the hottest and coldest 30 days), and they show a stronger impact from climate change (Figure S4 in Supporting Information S1). Numbers for all temperature‐related deaths at 3°C warming are tabulated in Section S6 in Supporting Information S1.

## 7 Conclusions

In this paper, we use mortality and temperature data obtained between 1987 and 2000 to develop a temperature‐mortality relationship for 106 cities in the U.S. covering about 65% of the total population. We then combine these regression models with temperatures from an ensemble of high‐resolution climate simulations to estimate future temperature‐related deaths. Because of the key role of adaptation, we make two different adaptation scenarios: a scenario with no adaptation and what we consider to be an aggressive adaptation scenario that follows the observed variations in adaptation between cities with different climates. We also incorporate estimates of changing population and its age distribution.

We estimate that there was an average of 36,444 temperature‐related deaths per year during the period 1987–2000 in the cities in our data set. Consistent with previous work (Berko, 2014; Gasparrini et al., 2015a, 2017; Heutel et al., 2021), we find that 86% of these deaths were cold‐related. Most of the cold‐related deaths took place at moderate temperatures just below the MMT, typically around 20–22°C, so they are categorized as cold related even though many would consider the temperatures to be mild.

We project that, with a warming climate and an increasing and aging population, temperature‐related deaths in these cities will reach 200,000 per year at 3°C of global average warming without adaptation. Adaptation reduces the increase of this number of temperature‐related deaths at 3°C of warming by 28%.

By decomposing mortality into climate, demographics, and population factors, we find that demographic shifts, primarily the aging of the population, and increasing population—will be the biggest drivers of increased temperature‐related mortality. Climate change will cause small changes in mortality below 3°C of global average warming due to offsetting decreases in cold‐related mortality and increases in heat‐related deaths. Above 3°C, the result depends on the level of adaptation with increases in heat‐related deaths dominating without adaptation. Without adaptation, total mortality rises rapidly; with adaptation, mortality declines. In our strong adaptation scenario, total heat‐related mortality decreases at all levels of warming.

The health impacts of extreme heat are just one of many health impacts of climate change (Rocque et al., 2021). As such, this research should not be interpreted as suggesting that climate change will yield net health benefits for low levels of warming.

While changes in temperature‐related mortality due to climate change may be small below 3°C, there is a meridional shift of mortality, with deaths shifting from the South to the North (Figures 5g and 5h). Since Southern cities in U.S. are already well adapted to heat, additional warming does not add a significant number of deaths. Northern cities, on the other hand, are not well adapted to heat, so heat‐related mortality increases there dominate decreases in cold‐related mortality.

Ultimately, no one knows how effectively we will adapt to the warmer temperatures of the coming century. However, the investments society has made to make cities like Houston or Phoenix livable in a hot climate are massive and it is far from assured that we will make similar investments in other cities as the climate warms. Many adaptive responses (e.g., installing air conditioning, improved health care, better urban planning) are too expensive for poorer individuals or communities, so adaptation will necessarily require society to pay for much of the adaptation. This would represent a huge transfer of wealth from richer to poorer members of our society, a dicey proposition in today's political environment.

There are important limitations to our analysis. Among the most important, our analysis covered 106 medium and large cities in the U.S., so we can't reach any conclusions about rural populations of the U.S. population or states that are not included in the mortality data set (MT, ID, WY, ND, and SD). Second, we cannot comment on the future of heat‐related mortality in the rest of the world. However, given the wealth of the U.S., our present levels of adaptation are higher than in many poorer countries and our ability to enhance our adaptation is also higher. Thus, it seems likely that heat‐related mortality will be a more significant problem in the rest of the world as climate change progresses through the century (Carleton et al., 2022). Finally, a limitation of our analysis arises from the temperature data set and projections used. We utilized 22 × 22 km boxes from the ERA‐5 temperature data set to represent the city's temperatures and sensitivity tests indicate that the size of the box we use introduces little error in our analysis. However, there are clear limitations to using this type of reanalysis for estimating the temperature of the city, in particular the neglect of the urban heat island effect. Future analysis should focus on using higher resolution temperature data in analyses to better capture this effect.

Nevertheless, this study integrates climate change, population and demographic change, and adaptation to project future temperature‐related deaths in the U.S. To our knowledge, only a handful of studies have attempted such a comprehensive approach, particularly emphasizing the range of adaptation. By doing so, our study highlights the importance of adaptation measures and identifies the most vulnerable cities in terms of future temperature‐related deaths, in addition to considering climate and population/demographic changes.

## Conflict of Interest

The authors declare no conflicts of interest relevant to this study.

## Supporting information

Supporting Information S1

## Data Availability

Mortality data from National Morbidity Mortality Air Pollution Study is available on R package: https://cran.r-project.org/src/contrib/Archive/NMMAPSlite/. Historical ERA‐5 temperature data are available on Climate Data Store: https://cds.climate.copernicus.eu/cdsapp#!/dataset/reanalysis-era5-land?tab=overview. NA‐CORDEX temperature data are available on Climate Data Gateway by NCAR: https://www.earthsystemgrid.org/search/cordexsearch.html. Future SSP population and demographics data are available in Socioeconomic Data and Applications Center (SEDAC): https://sedac.ciesin.columbia.edu/data/set/ssp-1-8th-urban-land-extent-projection-base-year-ssp-2000-2100.
