# Supplementary material for: Future Temperature‐Related Deaths in the U.S.: The Impact of Climate Change, Demographics, and Adaptation
Source: Geohealth. 2023 Aug 15;7(8):e2023GH000799. doi: 10.1029/2023GH000799 (PMC10426332; doi:10.1029/2023GH000799)
Supplement: Supplementary file 1 — Supporting Information S1 [file GH2-7-e2023GH000799-s001.docx]

*GeoHealth*

Supporting Information for

**Future Temperature Related Deaths in the US: the Impact of Climate Change and Adaptation**

Jangho Lee and Andrew E. Dessler

Department of Atmospheric Sciences, Texas A&M University, College Station, TX, USA

**Contents of this file**

Text S1 to S6

Figures S1 to S4

Tables S1 to S3

**S1. Distributed Lag Non-linear Model (DLNM) – Model Specification and Sensitivity**

The DLNM setup in this study follows a framework from previous studies Gasparrini et al. (2015a; 2015b). All calculations are done with the R packages *dlnm* and *mvmeta*.

*S1.1. First stage model*

In the first stage, the location and the age-specific temperature-mortality relationship is derived using a generalized linear model with a quasi-Poisson family. We use the following equation in this model:

$$\begin{aligned} \log\left( {Death}_{c,a} \right)=cb\left( {tMean}_{c}, lag=21 \right)+DOW+ns\left( DOY \right)+ns\left( Year \right)\#\left( 1 \right) \end{aligned}$$

Where ${Death}_{c,a}$ represents number of daily deaths in city *c* and age group *a*. $cb\left( {tMean}_{c}, lag=21 \right)$ is a cross basis function of temperature in city *c*, with up to 21 days of lag, which is obtained by the two equations of exposure-response relationship and lag-response relationship between temperature and mortality (Gasparrini 2014). In this study, we select a cross-basis composed of quadratic B-spline with three internal knots placed at the 10^th^, 75^th^, and 90^th^ percentiles of the location-specific temperature. An indicator of day of week (*DOW*) is included for the weekly cycle. A natural cubic B-spline with 8 degrees of freedom for day of year is included to control the seasonal cycle (*ns(DOY)*), and a natural cubic B-spline with 1 degree of freedom per decade is included for the long-term trend (*ns(Year)*).

The association of overall temperature-mortality relationship from eq. 1 is reduced to the cumulative relationship between temperature and mortality with the function *crossreduce*, included in *dlnm*.

*S1.2. Second stage model*

The multivariate meta-analysis model (Gasparrini and Armstrong 2013; Gasparrini et al. 2012) is used for the meta-analysis. It is difficult to extract the temperature-mortality relationship from some of the cities with small number of populations, due to high signal-to-noise ratio of daily deaths. Multivariate meta-analysis allows the temperature-mortality relationship in small cities to share the information of temperature-mortality relationship of larger cities with similar characteristics. For the characteristics for the city, we include average temperature, temperature range (75^th^ percentile – 25^th^ percentile), and latitude of each city (Gasparrini and Armstrong 2011; Gasparrini et al. 2015a; Gasparrini et al. 2015b). Package *mvmeta* is used for this analysis, and technical details of this analysis can be found in Gasparrini and Armstrong (2013).

*S1.3. Calculation of excess deaths due to temperature*

Cumulative risk ratio (RR) is calculated as a sum of RR in all lags (up to 21 days). This returns a cumulative RR relative to the mortality at minimum mortality temperature (MMT; Fig. 1 in main text and Fig. S1). Baseline deaths per thousand (baseline DPT) at the MMT is calculated by averaging the DPT values for the days within 0.5°C of MMT. From this, we can calculate DPT values at each day by multiplying cumulative RR to base DPT. We then calculate the number of excess deaths due to temperature by multiplying excess DPT by population.

*S1.4. Sensitivity analysis*

We tested the sensitivity of our results to the selection of parameters in the DLNM. The number of degrees of freedom to account for seasonality (dfSeas) was modulated from 7 to 9 (current value=8), and the number of degrees of freedom to account for the long-term trend (dfTrend) was modulated from 1 to 2 (current value=1). Table S1 shows the percent change of number of deaths caused by this modulation, calculated for each city. Overall, the choice of parameters changes excess deaths by less than 8%.

| JJA | | | |
| --- | --- | --- | --- |
|  | dfSeas=7 | dfSeas=8 | dfSeas=9 |
| dfTrend=1 | 1.76 (3.95) | 0 | 0.02 (3.21) |
| dfTrend=2 | 1.86 (4.10) | -0.08 (0.08) | -0.10 (3.26) |
|  |  |  |  |
| DJF | | | |
|  | dfSeas=7 | dfSeas=8 | dfSeas=9 |
| dfTrend=1 | 4.19 (8.84) | 0 | -7.55 (5.06) |
| dfTrend=2 | 4.81 (8.60) | 0.52 (0.84) | -6.92 (4.47) |

**Table S1.** Percent change of number of deaths due to sensitivity analysis. Percent changes are calculated for each city and average percent changes are shown in the table, while the inter-city standard deviation is shown in parentheses.

*S1.5. Impact of Ozone*

High Ozone (O_3_) concentration is known to impact human health (Ren et al. 2008). However, O3 is also known to be correlated with temperature, especially in summertime (Porter and Heald 2019), so it is difficult to distinguish the impact of O_3_ and temperature on number of deaths. In that context, we tested if prediction errors of the DLNM (residuals) correlated with O_3_ concentration.

In cities that average more than 20 daily deaths (24 cities), we calculate the prediction residual and regress against O_3_ concentration. Annually, the p value of this regression is 0.58 (inter-city standard deviation 1σ=0.28). For JJA, the p value is 0.48 (1σ=0.29), showing no significant correlation between the prediction residual and O_3_ concentration.

Since the effect of O_3_ could be non-linear, we computed the composite analysis between the residuals on the high O_3_ days (over 75^th^ percentile of O_3_) and low O_3_ days (under 25^th^ percentile of O_3_). In a t-test comparing the means of the annual values, the p value is 0.59 (1σ=0.27). When comparing only JJA, the p value is 0.48 (1σ=0.30), showing that there is no significant difference of the prediction residuals on high O_3_ days vs. low O_3_ days.

With this analysis, we see no evidence that our results are impacted by O_3_. However, given the high collinearity between temperature and O_3_, we cannot rule out some contribution to mortality from O_3_. Clearly, more work on this is warranted.

**S2. RR Curve for Populated Cities.**

Fig. 1 in main text shows the RR values that are averaged for all cities. Fig. S1 shows the RR curves for the 25 most populated cities.


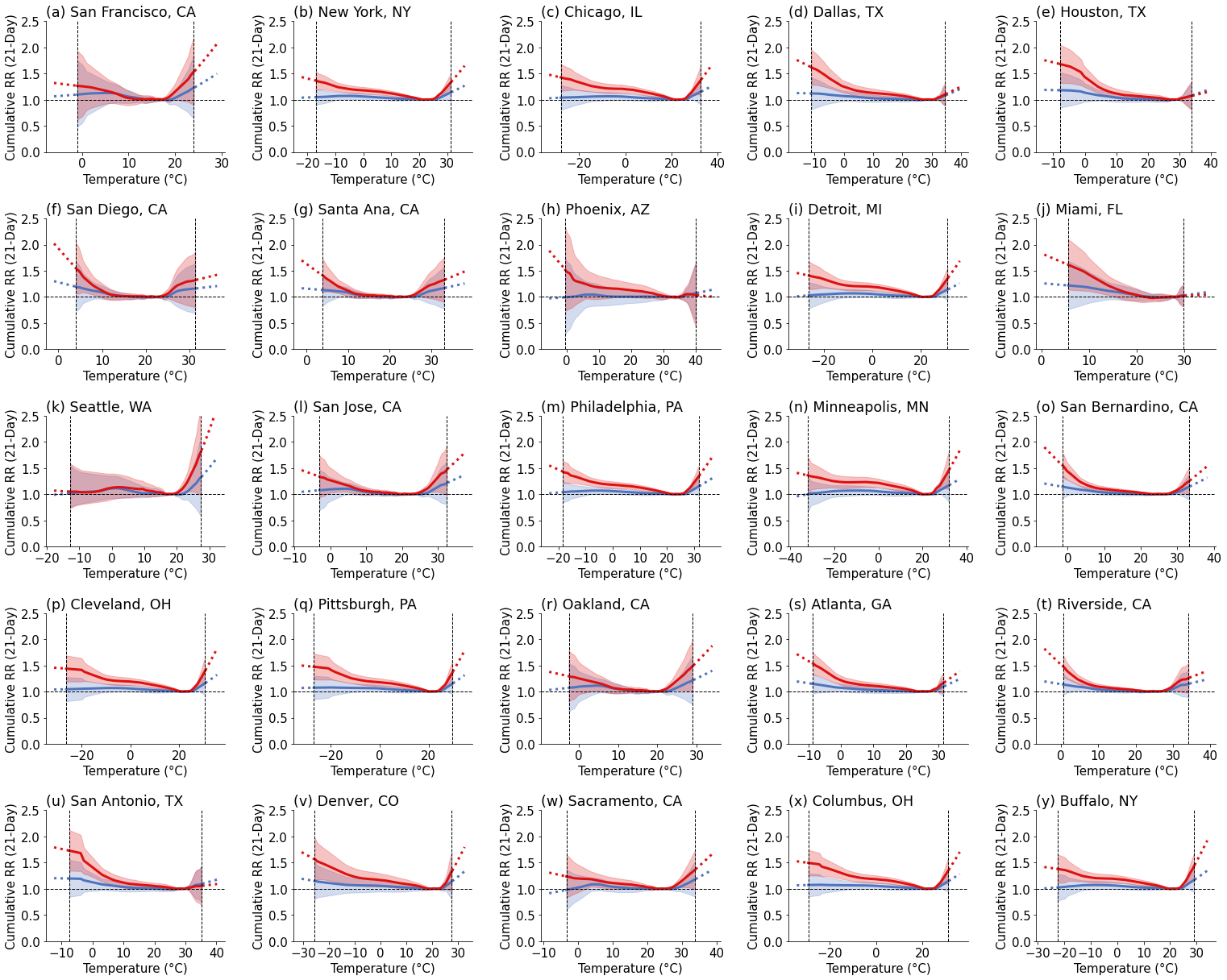


**Figure S1.** RR curve for 25 most populated cities. The red line represents the RR for the over 75 age group and the blue line represents the under 75 group. Solid lines are for historical temperature range, and dashed line are extrapolated RR values for the temperature outside the historical observations. Shaded regions show the 95% confidence interval of RR curve.

**S3. Measuring and Applying Adaptation – Example of New York**

For a more detailed explanation of measuring adaptation, here we go through an example of how we apply adaptation in our analysis. We select the >75 old age group in the city of New York City (NYC) in this example, but same process is applied for all age groups and all individual cities.

Fig. S2a shows that, in the 1987-2000 period, ERA-5 median JJA temperature in NYC was 22.7°C. From the projections of CORDEX-NA, median JJA temperature rises to 25.6°C in a world with 3°C global average warming.

In the 1987-2000 period, the hot-side RR slope of NY is 0.0491 (RR/°C/°C) (blue dashed line and blue dot in Fig. S2b). As seen in Fig. S2c, the RR curves are not linear, but the linear fit gives us a metric for how steeply the curve rises.

Using the slopes of the linear fit computed from all cities, we find that the hot side RR slope changes by -0.0057 (RR/°C/°C) as JJA median temperature increases (Fig. 3c in the main text, gray dashed line in Fig S2b). Using the increase in JJA median temperature for NYC, we therefore estimate that the hot-side RR slope of NY would decrease to 0.0296 (RR/°C) in a 3°C warmer world (red dashed line and red dot in Fig. S2b).

The last step is to adjust the RR curve by multiplying the hot-side mortality curve by the ratio of hot-side RR slope of 1987-2000 to that in 3°C world (0.0322/0.0491). This gives the RR curve in 3°C world (red line above the MMT in Fig. S2c). For the cold-side RR curve, the mortality curve is decreased by 18.5% of the ratio of the hot-side RR curve (red line below the MMT Fig. S2c), as discussed in the main text.


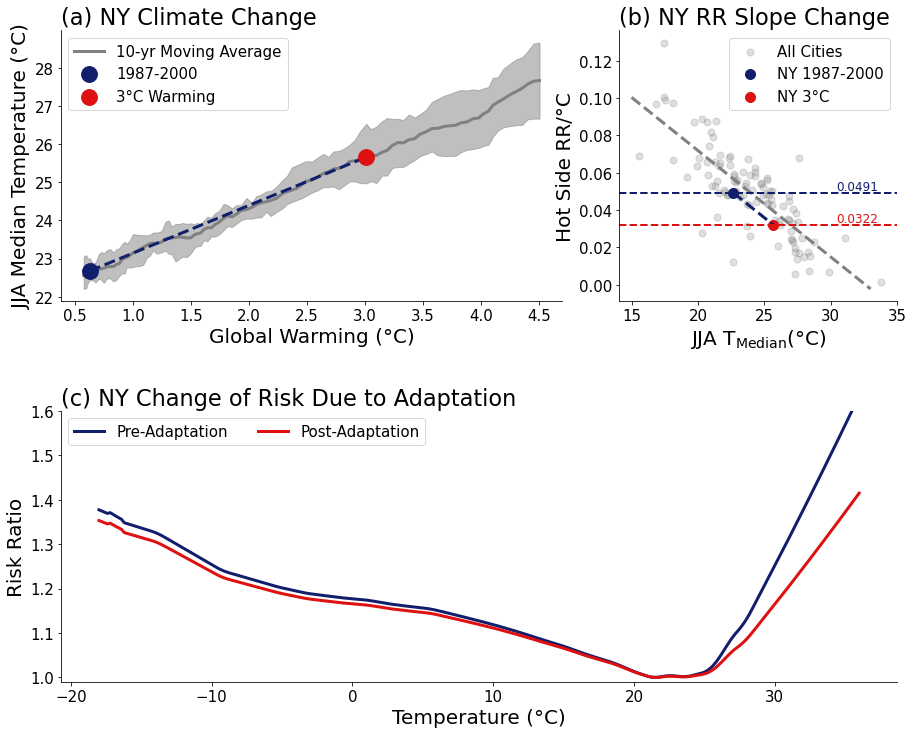


**Figure S2.** Example of measuring and applying adaptation, with >75 age group in New York City as example. (a) Current and future JJA median temperature. Gray shaded region is the upper and lower limit of climate projection from CORDEX-NA, and the gray solid line is the mean projection of NA-CORDEX. The values for the NA-CORDEX are smoothed with 10-yr moving average. Blue point is the 1987-2000 JJA median temperature from ERA-5, and red point is the JJA median temperature at 3°C of global warming. (b) Change of hot side RR slope with temperature. Gray points and dashed line represent the individual cities and the linear fit of those cities, same as Fig. 3c in the main text. Blue point and red point each show the hot side RR slope of NYC in 1987-2000 period and 3°C world. (c) Change of RR curve in NYC. Blue line represents the RR curve in 1987-2000 period, and red line represents the RR curve in 3°C world, when adaptation applied.

**S4. Future Population Scenario**

Fig. S3 summarizes the future population and demographic change. In the 106 cities used in this study, total population increases at a rate of 18.5 million/decade. The fraction of population over 75 increases at an average rate of 1.7%/decade. The top three cities with highest population increase are Austin (TX), Denver (CO), and Raleigh (NC), while top three cities with fastest aging population are Jackson (MS), Richmond (VA), and Santa Ana/Anaheim (CA).

Furthermore, we test the sensitivity due to future population scenario by comparing SSP2 (middle of the road) scenario with SSP5 scenario (currently used in the main text). First looking at total population, we observe a lower population increase in SSP2 scenario (Fig. S3a). This would decrease the contribution of population to total deaths (Figs. 4m-4p). The proportion of >75 age groups are very similar until year 2080 (3.3°C warming, Fig.S3b), so the contribution of changing demographics would be similar (Figs. 4i-4l), although the magnitude differs by the ratio of population in SSP5 and SSP2 (0.8 in 3°C warming). The impact of climate change (Figs. 4e-4h) is also similar with magnitude decreasing by the ratio of population in SSP5 and SSP2.

The inter-city pattern of slope of change in population and >75 age group ratio is nearly identical in SSP5 and SSP2. The R^2^ of the regression between the population slope distribution (Fig. S3c shows the slopes for SSP5) between SSP5 and SSP2 is 0.999 and R^2^ value of aging slope distribution (Fig. S3d) is 0.996.


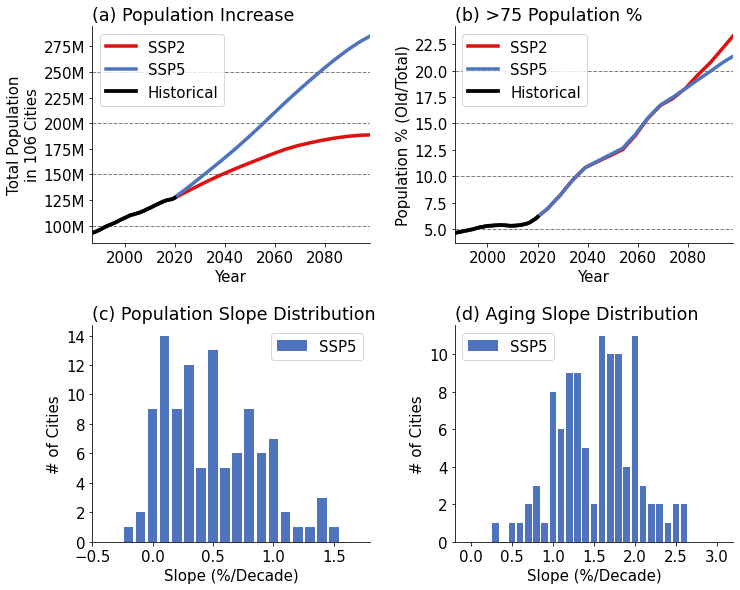


**Figure S3.** Summary of future population and demographic change. (a) Change of total population for all 106 cities in SSP2 and SSP5 scenarios. (b) Change in fraction of > 75 population, calculated by adding all > 75 population over 106 cities and dividing by total population. (c) Distribution of population trends of individual cities in the SSP5 scenario, relative to average historical population (1987-2020). (d) Distribution of growth of the fraction of the > 75 age group, in the SSP5 scenario.

**S5. Meridional Distribution of Extreme Temperature Related Deaths**

Here we show the plot same as Fig. 5, but using extreme temperature related deaths, which is deaths in 30 days each year with the highest and lowest temperatures. This shows a clear tendency for these deaths to occur at higher latitudes.


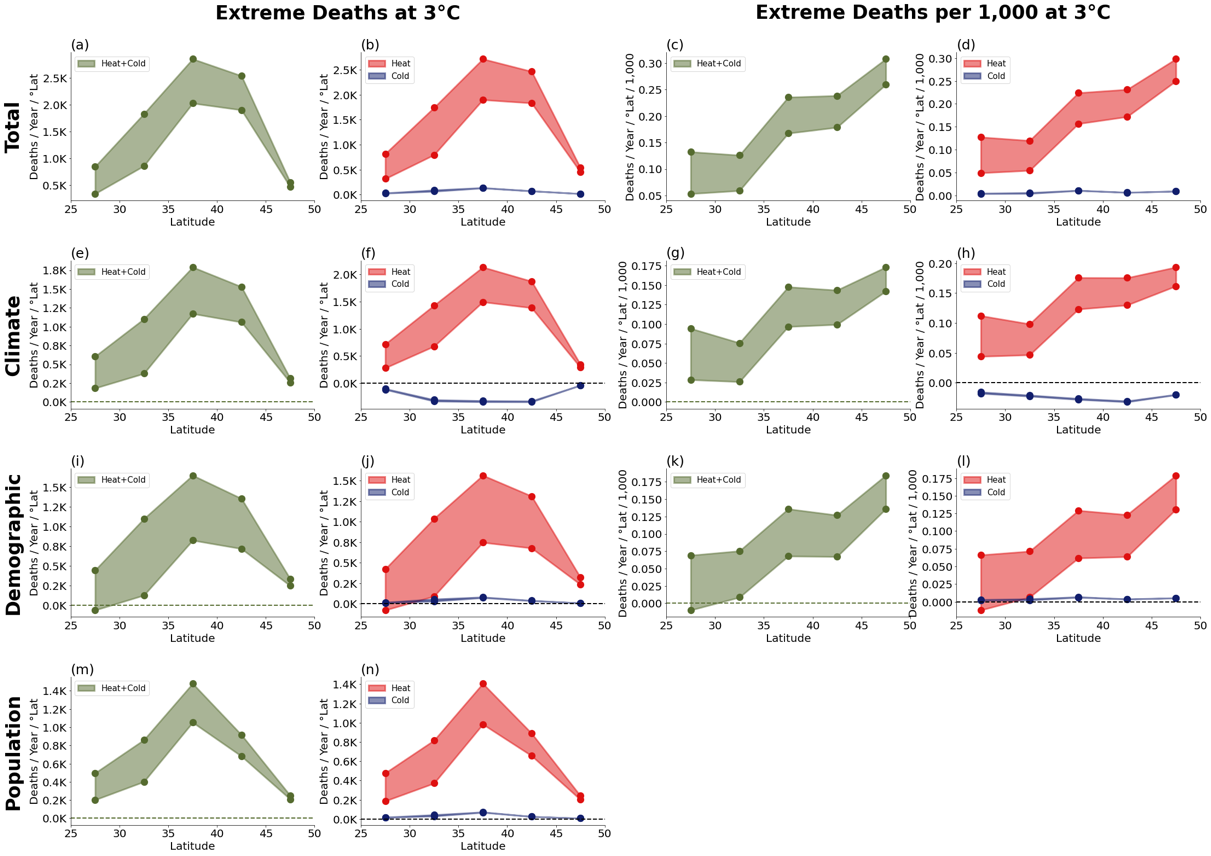


**Figure S4.** Meridional distribution of extreme temperature-related deaths in 3°C world, where extreme refers to the days with temperature higher than 97.5th percentile of 1987-2000 temperature (extreme heat) or below 2.5th percentile of temperature (extreme cold). (a) Number of deaths in 3°C world. Upper limit of the shaded region represents no-adaptation scenario, while the lower limit represents the adaptation scenario. (b) Same as (a), but for heat- and cold-related extreme deaths. (c, d) Same as (a, b), but per capita (each bin has been divided by population in that bin). (e-h) Contribution of climate change to mortality, (i-l) contribution of demographic changes to mortality, (m, n) contribution of changes in population.

**S6. Future predictions of Temperature Related Deaths**

Table S2 presents projected total mortality figures at a 3°C global average warming, considering all factors, as well as our estimates of deaths attributable solely to climate change. Additionally, the final column of Table S2 indicates each city's average warming relative to global warming in a 3°C warmer world.

We have also calculated the increase in annual average temperature for each city when the Earth reaches 3°C of warming, relative to the 2012-2022 period. We note that the global average warming over this period is 2°C, so most of the cities are warming more than the global average.

Similarly, Table S3 provides corresponding data for extreme temperatures, allowing a comprehensive understanding of both average and extreme warming scenarios for each city in the context of global climate change.

**Table S2.** The number of temperature related deaths in each city at 3°C warming, and number of deaths caused by climate change. XA = excluding adaptation, OA = with adaptation. Negative numbers indicate a reduction in mortality at 3°C. Shading in the table represents the magnitude of increase (red) and decrease (blue). Last column shows the city-specific warming at 3°C world, relative to global warming.

| **City** | **Deaths** | | | | | | **Climate Effect** | | | | | |  |
| --- | --- | --- | --- | --- | --- | --- | --- | --- | --- | --- | --- | --- | --- |
|  | **Heat+Cold** | | **Heat** | | **Cold** | | **Heat+Cold** | | **Heat** | | **Cold** | | **Warming** |
|  | **XA** | **OA** | **XA** | **OA** | **XA** | **OA** | **XA** | **OA** | **XA** | **OA** | **XA** | **OA** | **°C** |
| Akron, OH | 713 | 645 | 244 | 194 | 468 | 451 | 13 | -8 | 118 | 93 | -105 | -101 | 2.58 |
| Albuquerque, NM | 972 | 706 | 320 | 129 | 652 | 577 | -16 | -90 | 159 | 66 | -176 | -156 | 2.58 |
| Arlington, VA | 623 | 555 | 199 | 150 | 424 | 405 | -3 | -22 | 93 | 69 | -96 | -92 | 2.37 |
| Atlanta, GA | 3080 | 2633 | 972 | 653 | 2109 | 1980 | -75 | -215 | 546 | 368 | -621 | -583 | 2.25 |
| Austin, TX | 2255 | 1451 | 674 | 143 | 1581 | 1308 | -316 | -516 | 376 | 93 | -692 | -609 | 2.69 |
| Bakersfield, CA | 1200 | 971 | 491 | 311 | 709 | 660 | 64 | -20 | 271 | 173 | -207 | -193 | 2.16 |
| Baltimore, MD | 782 | 716 | 272 | 223 | 510 | 493 | 20 | -1 | 138 | 113 | -118 | -114 | 2.41 |
| Baton Rouge, LA | 309 | 231 | 113 | 55 | 195 | 176 | -17 | -42 | 68 | 35 | -85 | -77 | 2.32 |
| Biddeford, ME | 613 | 562 | 238 | 198 | 375 | 364 | 38 | 19 | 125 | 103 | -87 | -84 | 2.64 |
| Birmingham, AL | 612 | 496 | 207 | 123 | 404 | 373 | -1 | -41 | 124 | 74 | -125 | -115 | 2.32 |
| Boston, MA | 2214 | 1993 | 837 | 667 | 1376 | 1326 | 98 | 21 | 427 | 338 | -329 | -317 | 2.54 |
| Buffalo, NY | 1536 | 1413 | 567 | 472 | 969 | 940 | 87 | 43 | 293 | 243 | -207 | -200 | 2.69 |
| Cayce, SC | 413 | 330 | 141 | 81 | 272 | 250 | -10 | -34 | 72 | 42 | -82 | -75 | 2.24 |
| Cedar Rapids, IA | 648 | 565 | 203 | 144 | 445 | 421 | 2 | -20 | 92 | 65 | -90 | -85 | 2.84 |
| Charlotte, NC | 2658 | 2286 | 903 | 630 | 1754 | 1656 | 5 | -114 | 479 | 333 | -474 | -447 | 2.24 |
| Chicago, IL | 8665 | 7827 | 2531 | 1949 | 6134 | 5878 | -98 | -329 | 1213 | 926 | -1311 | -1255 | 2.80 |
| Cincinnati, OH | 1042 | 921 | 347 | 259 | 695 | 662 | 12 | -25 | 172 | 128 | -160 | -153 | 2.48 |
| Cleveland, OH | 1456 | 1348 | 468 | 390 | 988 | 958 | 15 | -18 | 235 | 195 | -220 | -213 | 2.66 |
| Columbus, GA | 305 | 259 | 113 | 78 | 192 | 181 | 8 | -9 | 70 | 49 | -62 | -58 | 2.22 |
| Columbus, OH | 3511 | 3126 | 1161 | 879 | 2351 | 2247 | 11 | -101 | 548 | 412 | -537 | -513 | 2.51 |
| Colorado Springs, CO | 1833 | 1653 | 907 | 756 | 926 | 897 | 316 | 225 | 603 | 502 | -286 | -277 | 2.76 |
| Corpus Christi, TX | 327 | 228 | 187 | 104 | 140 | 124 | 21 | -21 | 132 | 78 | -111 | -99 | 2.56 |
| Coventry, RI | 204 | 180 | 72 | 54 | 132 | 126 | 7 | -1 | 38 | 28 | -30 | -29 | 2.51 |
| Dayton, OH | 528 | 471 | 181 | 140 | 346 | 331 | 9 | -9 | 89 | 68 | -80 | -77 | 2.55 |
| Washington, DC | 2346 | 2109 | 772 | 599 | 1574 | 1509 | 8 | -61 | 371 | 286 | -363 | -348 | 2.37 |
| Denver, CO | 8438 | 7269 | 3648 | 2713 | 4790 | 4556 | 1093 | 564 | 2293 | 1706 | -1200 | -1142 | 2.69 |
| Des Moines, IA | 1443 | 1253 | 450 | 316 | 993 | 937 | 2 | -50 | 217 | 152 | -214 | -202 | 2.88 |
| Detroit, MI | 1555 | 1376 | 486 | 359 | 1069 | 1017 | 14 | -35 | 220 | 162 | -207 | -197 | 2.66 |
| Dallas/Fort Worth, TX | 7806 | 5467 | 2411 | 792 | 5395 | 4675 | -332 | -1071 | 1472 | 516 | -1804 | -1587 | 2.73 |
| El Paso, TX | 818 | 532 | 194 | 35 | 625 | 497 | -118 | -173 | 81 | 19 | -199 | -192 | 2.54 |
| Evansville, IN | 333 | 282 | 115 | 78 | 218 | 204 | 10 | -6 | 61 | 41 | -51 | -47 | 2.49 |
| Fresno, CA | 1472 | 1198 | 595 | 380 | 877 | 819 | 79 | -18 | 304 | 192 | -225 | -210 | 2.09 |
| Fort Wayne, IN | 816 | 706 | 277 | 195 | 540 | 511 | 4 | -26 | 124 | 87 | -120 | -113 | 2.64 |
| Grand Rapids, MI | 1835 | 1647 | 653 | 512 | 1182 | 1136 | 60 | 5 | 296 | 231 | -235 | -226 | 2.63 |
| Greensboro, NC | 981 | 862 | 348 | 259 | 633 | 603 | 13 | -25 | 180 | 134 | -167 | -159 | 2.27 |
| Houston, TX | 5956 | 3763 | 1985 | 460 | 3971 | 3303 | -770 | -1387 | 1154 | 298 | -1924 | -1685 | 2.48 |
| Huntsville, AL | 740 | 629 | 238 | 159 | 501 | 470 | -2 | -39 | 140 | 94 | -142 | -133 | 2.33 |
| Indianapolis, IN | 1770 | 1536 | 592 | 422 | 1177 | 1115 | 9 | -58 | 281 | 199 | -272 | -257 | 2.58 |
| Jackson, MS | 368 | 283 | 123 | 62 | 244 | 221 | -12 | -40 | 72 | 37 | -85 | -77 | 2.41 |
| Jacksonville, FL | 963 | 776 | 451 | 297 | 512 | 479 | 67 | -19 | 303 | 202 | -237 | -221 | 2.13 |
| Jersey City, NJ | 1622 | 1433 | 561 | 420 | 1061 | 1013 | 35 | -23 | 269 | 200 | -234 | -223 | 2.45 |
| Johnstown, PA | 89 | 81 | 33 | 27 | 56 | 55 | 4 | 1 | 16 | 13 | -13 | -12 | 2.43 |
| Kansas City, MO | 2175 | 1926 | 729 | 548 | 1446 | 1378 | 67 | -22 | 423 | 318 | -356 | -339 | 2.76 |
| Kansas City, KS | 324 | 287 | 111 | 84 | 213 | 203 | 13 | 0 | 66 | 50 | -53 | -50 | 2.76 |
| Kingston, NY | 231 | 213 | 84 | 70 | 147 | 143 | 8 | 2 | 40 | 33 | -32 | -31 | 2.50 |
| Knoxville, TN | 732 | 649 | 265 | 203 | 467 | 446 | 30 | -1 | 153 | 117 | -123 | -118 | 2.24 |
| Los Angeles, CA | 7391 | 4216 | 2581 | 369 | 4810 | 3848 | -1894 | -2435 | 1005 | 203 | -2900 | -2638 | 1.95 |
| Lafayette, LA | 439 | 304 | 160 | 59 | 279 | 244 | -29 | -72 | 95 | 38 | -125 | -109 | 2.33 |
| Las Vegas, NV | 6564 | 4997 | 1974 | 906 | 4591 | 4091 | 36 | -369 | 974 | 467 | -938 | -837 | 2.40 |
| Lexington, KY | 653 | 558 | 221 | 151 | 433 | 407 | 6 | -24 | 113 | 77 | -108 | -101 | 2.42 |
| Lincoln, NE | 779 | 646 | 246 | 153 | 533 | 493 | 10 | -28 | 125 | 78 | -115 | -107 | 2.83 |
| Lake Charles, LA | 261 | 181 | 93 | 35 | 168 | 146 | -18 | -43 | 59 | 24 | -77 | -68 | 2.41 |
| Louisville, KY | 1498 | 1286 | 498 | 343 | 1000 | 943 | 9 | -57 | 254 | 175 | -245 | -231 | 2.46 |
| Little Rock, AR | 569 | 452 | 175 | 93 | 394 | 359 | -3 | -41 | 105 | 57 | -108 | -98 | 2.49 |
| Lubbock, TX | 409 | 285 | 133 | 45 | 277 | 240 | -26 | -60 | 72 | 26 | -98 | -86 | 2.84 |
| Madison, WI | 1435 | 1279 | 447 | 336 | 988 | 942 | -7 | -45 | 188 | 141 | -195 | -186 | 2.75 |
| Memphis, TN | 1333 | 1147 | 462 | 325 | 870 | 822 | 51 | -20 | 286 | 201 | -235 | -221 | 2.49 |
| Miami, FL | 1365 | 468 | 979 | 221 | 386 | 246 | 195 | -391 | 785 | 189 | -590 | -580 | 1.97 |
| Milwaukee, WI | 1526 | 1349 | 460 | 335 | 1066 | 1014 | 18 | -38 | 237 | 170 | -218 | -207 | 2.90 |
| Minneapolis/St. Paul, MN | 4752 | 4320 | 1403 | 1105 | 3349 | 3216 | 34 | -73 | 610 | 480 | -576 | -553 | 2.98 |
| Mobile, AL | 302 | 227 | 119 | 62 | 183 | 165 | 2 | -28 | 80 | 42 | -77 | -70 | 2.31 |
| Modesto, CA | 728 | 638 | 299 | 227 | 429 | 411 | 33 | -1 | 161 | 122 | -128 | -123 | 1.95 |
| Muskegon, MI | 548 | 511 | 196 | 168 | 352 | 343 | 37 | 22 | 115 | 98 | -78 | -76 | 2.88 |
| Nashville, TN | 1553 | 1342 | 541 | 385 | 1012 | 957 | 44 | -31 | 313 | 223 | -268 | -254 | 2.42 |
| Newport News, VA | 255 | 220 | 100 | 72 | 155 | 147 | 10 | -3 | 54 | 39 | -45 | -42 | 2.38 |
| New Orleans, LA | 1532 | 1383 | 694 | 573 | 838 | 810 | 137 | 61 | 513 | 424 | -375 | -363 | 2.49 |
| Norfolk, VA | 327 | 287 | 129 | 97 | 198 | 189 | 14 | -1 | 72 | 55 | -58 | -55 | 2.40 |
| Newark, NJ | 2122 | 1876 | 735 | 551 | 1388 | 1324 | 47 | -29 | 353 | 264 | -306 | -292 | 2.45 |
| New York, NY | 18954 | 16245 | 6440 | 4439 | 12513 | 11805 | 381 | -458 | 3164 | 2168 | -2783 | -2626 | 2.49 |
| Oakland, CA | 1589 | 1389 | 865 | 689 | 725 | 700 | 13 | -65 | 450 | 356 | -436 | -421 | 1.73 |
| Oklahoma City, OK | 1615 | 1270 | 528 | 282 | 1087 | 988 | 6 | -109 | 318 | 175 | -312 | -284 | 2.79 |
| Olympia, WA | 642 | 584 | 289 | 241 | 353 | 343 | 21 | 3 | 116 | 96 | -95 | -92 | 1.72 |
| Omaha, NE | 1534 | 1315 | 475 | 322 | 1059 | 993 | 14 | -49 | 239 | 163 | -226 | -212 | 2.86 |
| Orlando, FL | 1423 | 1119 | 803 | 538 | 621 | 582 | 237 | 67 | 603 | 410 | -367 | -343 | 2.03 |
| Philadelphia, PA | 2795 | 2484 | 998 | 764 | 1797 | 1719 | 91 | -8 | 491 | 376 | -401 | -384 | 2.39 |
| Phoenix, AZ | 9961 | 1098 | 2139 | 360 | 7822 | 738 | -1076 | -7074 | 684 | 171 | -1760 | -7245 | 2.41 |
| Pittsburgh, PA | 1646 | 1501 | 572 | 463 | 1074 | 1037 | 32 | -13 | 275 | 221 | -243 | -234 | 2.43 |
| Portland, OR | 2855 | 2569 | 1270 | 1034 | 1585 | 1534 | 128 | 44 | 490 | 395 | -363 | -351 | 1.77 |
| Providence, RI | 971 | 842 | 348 | 250 | 623 | 592 | 36 | -8 | 178 | 127 | -142 | -135 | 2.51 |
| Raleigh, NC | 2992 | 2573 | 1070 | 755 | 1923 | 1818 | 9 | -120 | 537 | 380 | -528 | -499 | 2.28 |
| Richmond, VA | 1160 | 1015 | 420 | 311 | 739 | 704 | 16 | -29 | 205 | 152 | -189 | -180 | 2.32 |
| Riverside, CA | 2388 | 1842 | 972 | 537 | 1416 | 1305 | -123 | -288 | 492 | 280 | -615 | -569 | 1.98 |
| Rochester, NY | 1072 | 993 | 389 | 329 | 683 | 664 | 44 | 15 | 206 | 173 | -162 | -158 | 2.77 |
| Sacramento, CA | 2427 | 2091 | 932 | 670 | 1494 | 1420 | 29 | -81 | 452 | 320 | -423 | -401 | 1.87 |
| Salt Lake City, UT | 3578 | 3118 | 1438 | 1080 | 2140 | 2038 | 396 | 209 | 849 | 640 | -453 | -432 | 2.55 |
| San Antonio, TX | 2389 | 1291 | 725 | 170 | 1664 | 1120 | -437 | -848 | 380 | 116 | -817 | -964 | 2.66 |
| San Bernardino, CA | 2074 | 1789 | 775 | 556 | 1299 | 1233 | -102 | -186 | 396 | 288 | -498 | -474 | 2.07 |
| San Diego, CA | 2133 | 1385 | 1434 | 742 | 700 | 642 | 162 | -213 | 922 | 486 | -760 | -699 | 1.77 |
| San Francisco, CA | 535 | 452 | 426 | 347 | 108 | 105 | 125 | 76 | 279 | 225 | -154 | -150 | 1.69 |
| San Jose, CA | 1728 | 1516 | 691 | 522 | 1037 | 994 | -266 | -310 | 257 | 191 | -523 | -501 | 1.78 |
| Seattle, WA | 2931 | 2732 | 1436 | 1267 | 1495 | 1465 | 284 | 210 | 670 | 588 | -386 | -378 | 1.73 |
| Shreveport, LA | 298 | 219 | 93 | 38 | 205 | 181 | -16 | -40 | 56 | 24 | -72 | -64 | 2.58 |
| Spokane, WA | 1592 | 1418 | 668 | 530 | 924 | 888 | 199 | 139 | 331 | 266 | -132 | -127 | 2.10 |
| Santa Ana/Anaheim, CA | 2211 | 1671 | 1251 | 774 | 960 | 897 | -73 | -288 | 707 | 442 | -780 | -730 | 1.86 |
| St. Louis, MO | 710 | 627 | 231 | 172 | 479 | 455 | 13 | -14 | 124 | 92 | -111 | -106 | 2.61 |
| Stockton, CA | 933 | 829 | 404 | 320 | 529 | 509 | 35 | -4 | 211 | 166 | -176 | -170 | 1.87 |
| St. Petersburg, FL | 736 | 466 | 375 | 148 | 362 | 318 | 50 | -87 | 285 | 119 | -235 | -205 | 2.11 |
| Syracuse, NY | 611 | 549 | 236 | 188 | 375 | 361 | 29 | 8 | 113 | 89 | -84 | -81 | 2.67 |
| Tacoma, WA | 1706 | 1556 | 783 | 660 | 922 | 897 | 114 | 63 | 347 | 290 | -233 | -227 | 1.74 |
| Tampa, FL | 1407 | 986 | 756 | 396 | 651 | 590 | 178 | -48 | 572 | 308 | -394 | -356 | 2.08 |
| Toledo, OH | 505 | 439 | 162 | 115 | 343 | 325 | 3 | -15 | 72 | 51 | -70 | -66 | 2.63 |
| Topeka, KS | 225 | 193 | 78 | 55 | 147 | 138 | 9 | -3 | 45 | 32 | -37 | -35 | 2.80 |
| Tucson, AZ | 456 | 232 | 114 | 25 | 342 | 207 | -57 | -150 | 43 | 13 | -100 | -164 | 2.43 |
| Tulsa, OK | 1373 | 1104 | 480 | 282 | 893 | 822 | 47 | -55 | 301 | 178 | -254 | -234 | 2.75 |
| Wichita, KS | 871 | 684 | 287 | 152 | 583 | 531 | 22 | -44 | 169 | 90 | -147 | -134 | 2.84 |
| Worcester, MA | 1164 | 1035 | 396 | 301 | 768 | 735 | 24 | -15 | 181 | 136 | -158 | -151 | 2.50 |

**Table S3.** The number of extreme temperature related deaths in each city at 3°C warming, and number of extreme temperature related deaths caused by climate change.

| **City** | **Deaths** | | | | | | **Climate Effect** | | | | | |
| --- | --- | --- | --- | --- | --- | --- | --- | --- | --- | --- | --- | --- |
|  | **Heat+Cold** | | **Heat** | | **Cold** | | **Heat+Cold** | | **Heat** | | **Cold** | |
|  | **XA** | **OA** | **XA** | **OA** | **XA** | **OA** | **XA** | **OA** | **XA** | **OA** | **XA** | **OA** |
| Akron, OH | 129 | 103 | 125 | 99 | 4 | 4 | 78 | 59 | 96 | 76 | -18 | -18 |
| Albuquerque, NM | 239 | 103 | 230 | 95 | 9 | 8 | 128 | 39 | 158 | 65 | -30 | -26 |
| Arlington, VA | 97 | 74 | 92 | 69 | 5 | 5 | 56 | 38 | 73 | 55 | -18 | -17 |
| Atlanta, GA | 576 | 397 | 544 | 367 | 32 | 30 | 363 | 217 | 462 | 310 | -99 | -93 |
| Austin, TX | 401 | 122 | 367 | 94 | 34 | 28 | 210 | -14 | 322 | 82 | -112 | -96 |
| Bakersfield, CA | 324 | 208 | 314 | 199 | 10 | 9 | 214 | 129 | 238 | 150 | -23 | -22 |
| Baltimore, MD | 148 | 123 | 143 | 117 | 5 | 5 | 93 | 73 | 113 | 93 | -20 | -19 |
| Baton Rouge, LA | 74 | 39 | 71 | 36 | 3 | 3 | 52 | 21 | 63 | 32 | -12 | -10 |
| Biddeford, ME | 139 | 115 | 138 | 114 | 1 | 1 | 95 | 77 | 105 | 87 | -10 | -9 |
| Birmingham, AL | 132 | 80 | 125 | 75 | 6 | 6 | 90 | 47 | 108 | 64 | -19 | -17 |
| Boston, MA | 458 | 364 | 452 | 358 | 6 | 6 | 297 | 226 | 343 | 271 | -46 | -44 |
| Buffalo, NY | 339 | 283 | 332 | 277 | 6 | 6 | 214 | 171 | 253 | 209 | -39 | -38 |
| Cayce, SC | 78 | 46 | 75 | 43 | 3 | 3 | 50 | 25 | 60 | 35 | -10 | -9 |
| Cedar Rapids, IA | 100 | 72 | 97 | 69 | 3 | 3 | 61 | 39 | 75 | 53 | -14 | -13 |
| Charlotte, NC | 525 | 372 | 499 | 348 | 25 | 24 | 333 | 214 | 400 | 278 | -67 | -64 |
| Chicago, IL | 1267 | 987 | 1208 | 930 | 59 | 57 | 714 | 498 | 968 | 742 | -255 | -244 |
| Cincinnati, OH | 194 | 147 | 182 | 136 | 12 | 11 | 112 | 76 | 146 | 109 | -34 | -33 |
| Cleveland, OH | 245 | 205 | 236 | 196 | 9 | 9 | 145 | 114 | 191 | 158 | -45 | -44 |
| Columbus, GA | 76 | 54 | 73 | 51 | 3 | 3 | 53 | 35 | 62 | 43 | -9 | -8 |
| Columbus, OH | 611 | 469 | 575 | 434 | 36 | 35 | 340 | 234 | 450 | 339 | -110 | -105 |
| Colorado Springs, CO | 756 | 636 | 721 | 602 | 35 | 34 | 513 | 421 | 566 | 472 | -53 | -51 |
| Corpus Christi, TX | 157 | 91 | 154 | 88 | 3 | 2 | 112 | 61 | 127 | 74 | -15 | -14 |
| Coventry, RI | 42 | 31 | 41 | 31 | 1 | 1 | 27 | 19 | 32 | 24 | -5 | -4 |
| Dayton, OH | 99 | 77 | 95 | 73 | 5 | 4 | 60 | 43 | 74 | 57 | -15 | -14 |
| Washington, DC | 390 | 307 | 371 | 288 | 19 | 18 | 228 | 164 | 295 | 228 | -67 | -64 |
| Denver, CO | 2963 | 2248 | 2788 | 2081 | 175 | 166 | 1941 | 1403 | 2155 | 1607 | -214 | -204 |
| Des Moines, IA | 222 | 159 | 213 | 151 | 9 | 8 | 136 | 88 | 171 | 120 | -35 | -33 |
| Detroit, MI | 258 | 192 | 254 | 187 | 5 | 5 | 156 | 108 | 185 | 136 | -29 | -28 |
| Dallas/Fort Worth, TX | 1521 | 571 | 1416 | 480 | 105 | 92 | 895 | 120 | 1248 | 427 | -352 | -307 |
| El Paso, TX | 114 | 30 | 106 | 24 | 8 | 7 | 42 | -13 | 77 | 17 | -36 | -30 |
| Evansville, IN | 69 | 48 | 65 | 45 | 4 | 3 | 42 | 26 | 53 | 36 | -11 | -10 |
| Fresno, CA | 397 | 255 | 384 | 242 | 13 | 13 | 247 | 145 | 278 | 174 | -31 | -29 |
| Fort Wayne, IN | 142 | 101 | 138 | 97 | 4 | 4 | 80 | 51 | 102 | 71 | -21 | -20 |
| Grand Rapids, MI | 353 | 277 | 349 | 274 | 3 | 3 | 219 | 165 | 253 | 197 | -33 | -32 |
| Greensboro, NC | 208 | 157 | 199 | 148 | 9 | 9 | 130 | 91 | 154 | 115 | -25 | -24 |
| Houston, TX | 1283 | 371 | 1218 | 317 | 65 | 54 | 776 | 40 | 1042 | 267 | -266 | -227 |
| Huntsville, AL | 146 | 100 | 136 | 90 | 10 | 9 | 92 | 54 | 118 | 78 | -26 | -24 |
| Indianapolis, IN | 305 | 220 | 291 | 207 | 14 | 13 | 177 | 113 | 229 | 162 | -52 | -49 |
| Jackson, MS | 76 | 40 | 72 | 36 | 4 | 3 | 52 | 21 | 63 | 32 | -12 | -11 |
| Jacksonville, FL | 330 | 220 | 323 | 214 | 7 | 7 | 262 | 168 | 283 | 188 | -21 | -19 |
| Jersey City, NJ | 310 | 233 | 302 | 225 | 8 | 8 | 183 | 127 | 225 | 167 | -42 | -40 |
| Johnstown, PA | 19 | 15 | 18 | 15 | 1 | 1 | 12 | 9 | 14 | 11 | -2 | -2 |
| Kansas City, MO | 419 | 322 | 394 | 298 | 25 | 24 | 264 | 185 | 336 | 253 | -71 | -68 |
| Kansas City, KS | 66 | 51 | 62 | 47 | 4 | 4 | 42 | 30 | 53 | 40 | -11 | -10 |
| Kingston, NY | 46 | 38 | 45 | 37 | 1 | 1 | 29 | 23 | 33 | 28 | -5 | -5 |
| Knoxville, TN | 173 | 134 | 162 | 124 | 11 | 10 | 112 | 80 | 136 | 104 | -24 | -23 |
| Los Angeles, CA | 879 | 212 | 824 | 167 | 55 | 45 | 385 | -101 | 678 | 149 | -293 | -249 |
| Lafayette, LA | 107 | 44 | 103 | 39 | 5 | 4 | 71 | 18 | 89 | 34 | -18 | -16 |
| Las Vegas, NV | 1373 | 665 | 1334 | 629 | 40 | 36 | 802 | 331 | 924 | 439 | -122 | -109 |
| Lexington, KY | 126 | 89 | 116 | 79 | 10 | 10 | 70 | 41 | 96 | 66 | -26 | -24 |
| Lincoln, NE | 131 | 85 | 124 | 79 | 6 | 6 | 81 | 45 | 100 | 63 | -19 | -18 |
| Lake Charles, LA | 64 | 27 | 61 | 25 | 3 | 2 | 43 | 12 | 54 | 22 | -11 | -10 |
| Louisville, KY | 286 | 203 | 261 | 180 | 24 | 23 | 154 | 90 | 215 | 148 | -61 | -57 |
| Little Rock, AR | 106 | 60 | 98 | 53 | 8 | 7 | 66 | 27 | 87 | 47 | -21 | -20 |
| Lubbock, TX | 86 | 34 | 80 | 29 | 6 | 5 | 46 | 7 | 64 | 23 | -18 | -16 |
| Madison, WI | 216 | 163 | 210 | 158 | 5 | 5 | 121 | 84 | 151 | 113 | -30 | -29 |
| Memphis, TN | 305 | 218 | 289 | 203 | 16 | 15 | 203 | 130 | 252 | 177 | -49 | -47 |
| Miami, FL | 764 | 185 | 756 | 180 | 8 | 5 | 628 | 128 | 670 | 165 | -42 | -37 |
| Milwaukee, WI | 258 | 188 | 251 | 181 | 7 | 7 | 163 | 108 | 203 | 146 | -40 | -38 |
| Minneapolis/St. Paul, MN | 746 | 590 | 732 | 577 | 13 | 13 | 431 | 322 | 529 | 416 | -98 | -94 |
| Mobile, AL | 85 | 46 | 83 | 43 | 2 | 2 | 65 | 30 | 74 | 38 | -9 | -8 |
| Modesto, CA | 178 | 136 | 172 | 130 | 6 | 6 | 117 | 84 | 136 | 102 | -19 | -18 |
| Muskegon, MI | 126 | 108 | 123 | 105 | 2 | 2 | 88 | 73 | 102 | 87 | -14 | -14 |
| Nashville, TN | 348 | 254 | 321 | 229 | 26 | 25 | 211 | 136 | 273 | 194 | -62 | -59 |
| Newport News, VA | 60 | 44 | 58 | 42 | 2 | 2 | 39 | 27 | 46 | 33 | -7 | -6 |
| New Orleans, LA | 516 | 428 | 506 | 418 | 10 | 9 | 434 | 354 | 472 | 390 | -37 | -36 |
| Norfolk, VA | 79 | 60 | 76 | 58 | 3 | 3 | 52 | 38 | 61 | 46 | -8 | -8 |
| Newark, NJ | 408 | 308 | 397 | 297 | 11 | 10 | 242 | 169 | 295 | 220 | -53 | -51 |
| New York, NY | 3642 | 2520 | 3546 | 2430 | 96 | 90 | 2168 | 1345 | 2666 | 1815 | -498 | -470 |
| Oakland, CA | 456 | 363 | 450 | 356 | 6 | 6 | 319 | 246 | 357 | 282 | -38 | -36 |
| Oklahoma City, OK | 324 | 185 | 302 | 165 | 22 | 20 | 205 | 91 | 263 | 144 | -58 | -53 |
| Olympia, WA | 168 | 141 | 163 | 136 | 6 | 5 | 88 | 72 | 101 | 84 | -12 | -12 |
| Omaha, NE | 246 | 171 | 234 | 160 | 12 | 11 | 154 | 96 | 190 | 129 | -36 | -34 |
| Orlando, FL | 650 | 442 | 636 | 428 | 14 | 13 | 543 | 358 | 579 | 391 | -36 | -34 |
| Philadelphia, PA | 567 | 436 | 553 | 423 | 14 | 14 | 346 | 251 | 408 | 311 | -63 | -60 |
| Phoenix, AZ | 1175 | 241 | 1086 | 233 | 89 | 7 | 391 | -117 | 634 | 155 | -243 | -272 |
| Pittsburgh, PA | 304 | 249 | 289 | 234 | 15 | 15 | 182 | 139 | 229 | 185 | -47 | -46 |
| Portland, OR | 749 | 611 | 723 | 586 | 26 | 25 | 395 | 310 | 447 | 360 | -52 | -50 |
| Providence, RI | 196 | 141 | 193 | 138 | 3 | 3 | 127 | 86 | 147 | 105 | -20 | -19 |
| Raleigh, NC | 590 | 421 | 568 | 400 | 23 | 21 | 378 | 248 | 451 | 317 | -73 | -69 |
| Richmond, VA | 227 | 170 | 217 | 161 | 10 | 10 | 137 | 94 | 170 | 125 | -33 | -31 |
| Riverside, CA | 510 | 287 | 491 | 269 | 19 | 18 | 317 | 149 | 391 | 217 | -74 | -68 |
| Rochester, NY | 212 | 179 | 208 | 175 | 4 | 4 | 137 | 111 | 166 | 140 | -30 | -29 |
| Sacramento, CA | 501 | 361 | 485 | 346 | 15 | 15 | 323 | 217 | 370 | 262 | -47 | -44 |
| Salt Lake City, UT | 1162 | 885 | 1113 | 839 | 48 | 46 | 708 | 513 | 816 | 616 | -108 | -103 |
| San Antonio, TX | 438 | 146 | 401 | 121 | 37 | 26 | 205 | -15 | 337 | 103 | -132 | -117 |
| San Bernardino, CA | 424 | 308 | 409 | 294 | 15 | 14 | 269 | 182 | 324 | 234 | -55 | -52 |
| San Diego, CA | 908 | 469 | 902 | 464 | 5 | 5 | 717 | 350 | 775 | 403 | -58 | -53 |
| San Francisco, CA | 295 | 239 | 294 | 238 | 1 | 1 | 233 | 187 | 241 | 194 | -7 | -7 |
| San Jose, CA | 256 | 195 | 234 | 173 | 23 | 22 | 101 | 56 | 184 | 135 | -82 | -79 |
| Seattle, WA | 918 | 811 | 894 | 788 | 24 | 23 | 538 | 467 | 591 | 519 | -53 | -52 |
| Shreveport, LA | 52 | 24 | 49 | 21 | 3 | 3 | 32 | 8 | 44 | 19 | -12 | -11 |
| Spokane, WA | 461 | 370 | 447 | 357 | 14 | 13 | 275 | 217 | 303 | 244 | -28 | -26 |
| Santa Ana/Anaheim, CA | 662 | 410 | 653 | 402 | 9 | 8 | 489 | 283 | 554 | 344 | -65 | -61 |
| St. Louis, MO | 125 | 95 | 119 | 88 | 7 | 7 | 77 | 53 | 99 | 73 | -21 | -20 |
| Stockton, CA | 227 | 180 | 221 | 174 | 6 | 6 | 155 | 118 | 175 | 137 | -20 | -19 |
| St. Petersburg, FL | 314 | 131 | 303 | 122 | 11 | 10 | 248 | 86 | 278 | 113 | -31 | -27 |
| Syracuse, NY | 130 | 103 | 127 | 101 | 2 | 2 | 80 | 60 | 95 | 75 | -15 | -15 |
| Tacoma, WA | 483 | 408 | 465 | 391 | 18 | 17 | 267 | 218 | 304 | 255 | -37 | -36 |
| Tampa, FL | 640 | 345 | 622 | 329 | 18 | 16 | 515 | 257 | 562 | 299 | -46 | -42 |
| Toledo, OH | 84 | 60 | 83 | 58 | 2 | 2 | 49 | 32 | 60 | 42 | -10 | -10 |
| Topeka, KS | 46 | 33 | 44 | 31 | 2 | 2 | 29 | 19 | 36 | 26 | -7 | -6 |
| Tucson, AZ | 60 | 18 | 56 | 16 | 3 | 2 | 25 | 0 | 37 | 11 | -13 | -12 |
| Tulsa, OK | 304 | 186 | 287 | 170 | 17 | 16 | 200 | 102 | 250 | 148 | -49 | -46 |
| Wichita, KS | 178 | 99 | 168 | 90 | 10 | 9 | 113 | 50 | 141 | 75 | -28 | -25 |
| Worcester, MA | 205 | 155 | 201 | 152 | 3 | 3 | 127 | 90 | 149 | 111 | -22 | -21 |

**Reference**

Gasparrini, A., 2014: Modeling exposure–lag–response associations with distributed lag non‐linear models. *Statistics in medicine*, **33,** 881-899.

Gasparrini, A., and B. Armstrong, 2011: The impact of heat waves on mortality. *Epidemiology (Cambridge, Mass.)*, **22,** 68.

——, 2013: Reducing and meta-analysing estimates from distributed lag non-linear models. *BMC medical research methodology*, **13,** 1-10.

Gasparrini, A., B. Armstrong, and M. G. Kenward, 2012: Multivariate meta‐analysis for non‐linear and other multi‐parameter associations. *Statistics in medicine*, **31,** 3821-3839.

Gasparrini, A., and Coauthors, 2015a: Mortality risk attributable to high and low ambient temperature: a multicountry observational study. *The lancet*, **386,** 369-375.

Gasparrini, A., and Coauthors, 2015b: Temporal variation in heat–mortality associations: a multicountry study. *Environmental health perspectives*, **123,** 1200-1207.

Porter, W. C., and C. L. Heald, 2019: The mechanisms and meteorological drivers of the<? xmltex\break?> summertime ozone–temperature relationship. *Atmospheric Chemistry and Physics*, **19,** 13367-13381.

Ren, C., G. M. Williams, L. Morawska, K. Mengersen, and S. Tong, 2008: Ozone modifies associations between temperature and cardiovascular mortality: analysis of the NMMAPS data. *Occupational and environmental medicine*, **65,** 255-260.
